# Influences of demographic, seasonal, and social factors on automated touchscreen computer use by rhesus monkeys (*Macaca mulatta*) in a large naturalistic group

**DOI:** 10.1371/journal.pone.0215060

**Published:** 2019-04-24

**Authors:** Regina Paxton Gazes, Meredith C. Lutz, Mark J. Meyer, Thomas C. Hassett, Robert R. Hampton

**Affiliations:** 1 Department of Psychology, Bucknell University, Lewisburg, Pennsylvania, United States of America; 2 Program in Animal Behavior, Bucknell University, Lewisburg, Pennsylvania, United States of America; 3 Department of Mathematics, Bucknell University, Lewisburg, Pennsylvania, United States of America; 4 Department of Mathematics and Statistics, Georgetown University, Washington, District of Columbia, United States of America; 5 Department of Psychology, Emory University, Atlanta, Georgia, United States of America; 6 Yerkes National Primate Research Center, Atlanta, Georgia, United States of America; Consiglio Nazionale delle Ricerche, ITALY

## Abstract

Animals housed in naturalistic social groups with access to automated cognitive testing vary in whether and how much they participate in cognitive testing. Understanding how demographic, seasonal, and social factors relate to participation is essential to evaluating the usefulness of these systems for studying cognition and in assessing the data produced. We evaluated how sex, age, reproductive experience, seasonality, and rank related to patterns of participation in a naturalistic group of rhesus monkeys over a 4-year period. Females interacted with the touchscreen systems more than males and were more likely to complete initial training. Age was positively correlated with touchscreen activity through adolescence in females, at which point seasonality and reproductive experience were stronger associates of participation. While monkeys in different rank categories did not differ in how much they interacted with the touchscreen systems, monkeys of different ranks tended not to work at the same times, perhaps reflecting avoidance of high ranking animals by those of lower rank. Automated cognitive testing systems for naturalistic social groups of rhesus monkeys can yield quality cognitive data from individuals of all ages and ranks, but participation biases may make it difficult to study sex differences or seasonal variation in cognition.

## Introduction

Decades of carefully controlled research with animals housed singly or in small groups has produced an essential base of knowledge on the mechanisms underlying cognition [1, 2]. In many nonhuman primate species, cognitive processes such as learning and memory evolved and function in complex social contexts. Recently there has been increased interest in understanding how these cognitive processes function in social settings and how social factors relate to cognitive function [3, 4]. Testing cognition in naturalistic social groups allows researchers to pose a broad array of research questions not possible in traditional research settings, including whether there is a relationship between dominance rank and cognitive processes [5], what animals know about their social relationships [6], and whether cognition relates to reproductive success [7].

Advances in technology have expanded opportunities for tests of cognition in animals living in naturalistic social groups [3, 8–12]. Radio frequency identification (RFID) chips allow computers to automatically identify individual animals, present them with individualized cognitive tasks, and record data by subject. These types of automated cognitive testing systems have been successfully implemented for socially-housed captive animals including guinea baboons (*Papio papio*) [13, 14], rhesus macaques [15], and bonnet macaques (*Macaca radiata*) [16], and in wild Carolina wrens (*Thryothorus ludovicianus*), great tits (*Parus major*), chickadees (*Poecile atricapillus)*[17–19].

In addition to providing the opportunity to investigate questions about social influences on cognition, automated testing of animals in naturalistic social groups offers some practical advantages. First, automated testing may provide benefits for the physical health and psychological well-being of research animals. Cognitive testing in naturalistic social groups has been associated with decreased cortisol levels, decreased stereotypies, and increased social behavior [20]. Second, automated testing in naturalistic social groups is cost effective and does not require experimenter intervention to separate and handle individual animals [21]. Finally, animals tested in social settings may be more motivated to participate in cognitive testing than animals tested individually [22].

Automated testing in naturalistic social groups produces cognitive performance similar to that produced by animals tested individually in the laboratory. Baboons and rhesus monkeys living in social groups with access to automated computerized testing systems learned complex cognitive tasks including motor control, visual perception, matching-to-sample, and same/different discriminations [14]. Rhesus monkeys tested in a large a social group did not differ from those tested individually in tests of perceptual function, classification, memory, or transitive inference [15]. Wild great tits show similar learning curves to captive, individually-tested birds on a spatial reversal learning task [23]. This body of evidences suggests that automated testing in naturalistic captive groups and in the wild can produce quality cognitive data.

A challenge in using automated cognitive testing systems in naturalistic social groups is that subjects have a larger range of possible alternative activities than would be the case for most laboratory-housed animals. For example, animals may have large numbers of possible social partners, providing many opportunities to engage in social interactions rather than engaging in cognitive testing. Species-specific social factors may therefore have a strong influence on both which animals participate in cognitive testing and when individuals participate. If demographic factors such as age, sex, or dominance rank affect participation in cognitive testing, it may be difficult to achieve sufficient sample size for some comparisons. For example if participation is higher in one sex, this makes between sex comparisons difficult. Additionally the individuals represented in group data might not be representative of the group as a whole [24]. Self-selection is of particular concern in species or in tasks in which these same demographic factors are related to cognitive performance. For example, in water maze tasks male rats outperform females while female mice outperform males [25]. If male rodents were more likely to participate in cognitive testing than females, that would bias estimates of average performance in these two species, inflating performance in rats and underestimating performance in mice. Sex, age, and rank have all been associated with both participation in cognitive tasks and with cognitive performance [13, 17, 24–29]. Finally, studies aimed at comparing subjects across variables such as sex, age, rank, and season require robust samples. Understanding how demographic factors relate to participation in cognitive testing is therefore essential for planning and interpreting studies in naturalistic social groups [23].

Below, we provide a brief review of literature on the effects of sex, age, social rank, season, reproductive experience, and social relationships on behavior and cognitive testing. We then present analyses of cognitive participation by monkeys housed in a naturalistic social group over a four-year period, focusing on these same demographic variables. Finally, we discuss the implications of our findings for planning cognitive studies in naturalistic social groups of rhesus monkeys.

### Sex

Female primates generally spend more time foraging than males [30–33], possibly due to increased caloric needs related to nursing and infant care [34]. As correct responses to cognitive tests result in a food reward, females may also be more likely to participate in cognitive testing. Analyses of the influence of sex on participation in automated cognitive testing for socially-living animals in other species have been mixed. Socially-housed male guinea baboons participated more than did females [13], but there were no sex differences in participation in wild great tits [23]. If differences in primate foraging apply to cognitive testing, we should observe females interacting with the touchscreen systems more than males.

### Age

Younger animals are often less neophobic than adults [17, 35]. This may lead younger animals to interact more with cognitive testing systems. Age was negatively correlated with use of automated cognitive testing systems in great tits and baboons [13, 23]. If younger animals are more likely to participate in cognitive testing regardless of species, then we should observe a negative relationship between age and touchscreen usage in rhesus macaques.

### Social rank

High ranking rhesus monkeys have priority access to preferred food, and low ranking monkeys sustain high levels of aggression when co-feeding with high ranking animals [36]. Dominance may result in high ranking monkeys monopolizing touchscreen systems to the exclusion of low ranking monkeys. A high ranking female guinea baboon inhibited use of a touchscreen testing system by lower ranking females [14]. If high ranking rhesus monkeys behave similarly, low ranking monkeys will be less likely to interact with the touchscreen systems than high ranking monkeys.

It is also possible that high and low ranking monkeys may not differ in engagement with the touchscreen systems, but rank may dictate when during the day monkeys engage with the touchscreens and whether they are equally likely to work with all other individuals. In general, low ranking monkeys time their foraging to minimize conflict with higher ranking animals. For example, low ranking female crab-eating macaques increase their food intake by arriving at a feeding site before higher ranking animals [37]. As with other resources, high-ranking animals may have priority access to the cognitive testing systems while low ranking monkeys may have to modulate their activity to minimize receiving aggression. In guinea baboons with access to an automated cognitive testing system, a low-ranking female interacted with the test systems at times of the day when the high-ranking female showed low activity, indicating that low-ranking animals might adaptively time cognitive testing to avoid conflict [14]. If rhesus monkeys partition their cognitive testing by social rank, low-ranking monkeys would be more likely to interact with the testing systems when high-ranking monkeys are not present.

### Season and reproductive experience

In seasonally-breeding animals such as rhesus monkeys [38], activity budgets and social behavior vary across the breeding cycle [39, 40]. For example, female vervet monkeys (*Chlorocebus pygerythrus*) spend significantly less time foraging during the birthing season than during other times of the year [40], presumably due to the need to engage in infant care instead. Time constraints associated with seasonal changes in mating and care of offspring may therefore result in decreased participation in cognitive testing during breeding seasons (birthing [spring] and mating [fall] seasons), at least for females. This may additionally result in decreased activity from females that have offspring, as they would be actively engaged in mating or care of offspring.

### Social relationships

Rhesus monkeys tend to affiliate with monkeys of the same sex, rank, and family group [41–43]. In naturalistic social groups in which multiple animals can participate in testing simultaneously, these preferences may extend to cognitive testing. For example, animals may prefer to participate in cognitive testing at the same time as kin or same sex individuals, and may avoid the test systems when a higher ranked individual is present. Indeed, baboons use touchscreen systems located near systems in use by preferred social partners [44]. If the demographic factors that are predictive of social partners also extend to co-working on cognitive tasks, we should observe monkeys engaging with the touchscreen systems at the same time as kin and same sex, same rank individuals.

We assessed the extent to which the above factors related to participation in automated cognitive testing in a naturalistic group of rhesus monkeys by analyzing four years of data from four automated computerized cognitive testing systems mounted in the home enclosure. We analyzed how sex, age, rank, seasonality, reproductive experience, and social relationships were related to *whether* individual monkeys participated in cognitive testing (Study 1) and, for monkeys who did participate, *how much*, *when*, *and with whom* they interacted with the cognitive testing systems (Study 2).

## General methods

All procedures were approved by the Institutional Animal Care and Use Committee of Emory University (Protocol numbers YER-2001135-090212GA and YER-2002015-081515GA) and complied with the National Institutes of Health guidelines for the care and use of laboratory animals.

### Subjects and housing

The study took place in a large, multi-male/multi-female group of rhesus macaque monkeys (*Macaca mulatta*) housed in a 30 X 30 m outdoor enclosure, connected with a temperature-controlled indoor area, at the Yerkes National Primate Research Center in Lawrenceville, GA. Over the study period, the group varied from 2–3 adult males that had been transferred into the group as adults, 36–64 adult females that had mostly been born into the group, and 28–37 juvenile males and females that had been born into the group. The total number of animals in the group ranged from 67 to 94 (M = 76.27) due to births, deaths, and removal of animals for colony management purposes. Over the course of the study period, a total of 128 monkeys (88 females, 40 males) were present in the group for long enough to interact with the cognitive testing systems. Monkeys ranged in age from 2 to 215 months over the course of the study. The median age at which monkeys received their RFID chip and could therefore access the touchscreen, hereafter *age at training*, was 13.65 months (interquartile range 48.55). For colony management purposes, and consistent with the pattern of male dispersal in nature [45] most males born in the group were removed at puberty (approximately 3 years of age). Food and water were available *ad libitum*. The primary diet of monkey chow (LabDiet Monky Diet 5037) was replenished daily at approximately 7:30 am and a scatter feeding of fruits and vegetables was provided at approximately 2 pm.

The monkeys showed mating and birthing patterns consistent with seasonal breeding, with most mating taking place from September to November, and 87% births occurring in the spring, between March and May. Twelve percent of births occurred later, between June and August, and just 1% of births occurred in fall or winter. Animals were retained for future research at the end of this study.

### Apparatus

**Fig 1:**
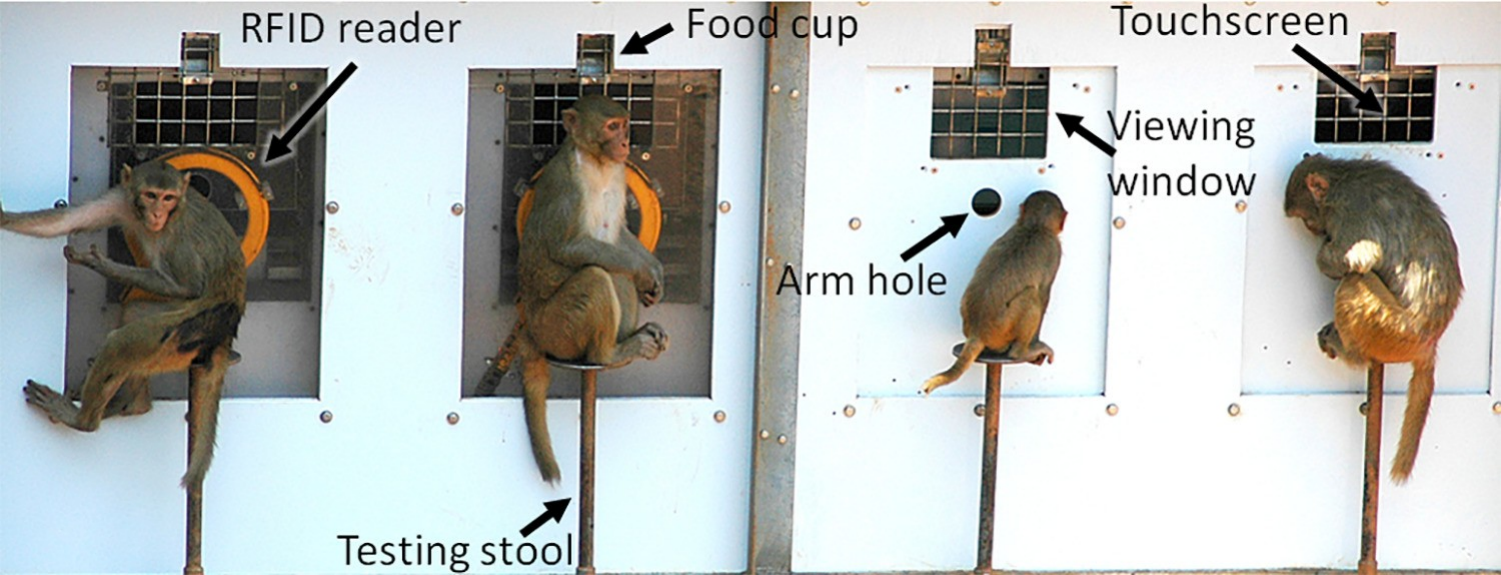
Four automated cognitive testing units were built into the wall of the monkey enclosure. Monkeys sat on a stool and reached through a hole to access the touchscreen, causing an RFID reader to identify the animal, via an antenna that surround the hole (the orange ring visible in the left two stations). Monkeys viewed the images presented on the touchscreen through a mesh window. Food rewards were delivered into the food cup positioned immediately above the window. Testing stations on the left had clear panels while testing stations on the right had opaque panels. Otherwise, the testing systems were identical.

Four touchscreen computer stations were built into the wall of the outdoor enclosure. Each station included a 15-inch LCD color monitor (3M, St. Paul, MN) running at a resolution of 1024 x 768 pixels, an automated pellet dispenser (Med Associates Inc., St. Albans, VT), stereo speakers, an RFID chip reader (Biomark, Boise, ID), and a stool for monkeys to sit on while working (Fig 1). Touchscreens were located 15 cm behind a poly panel in an enclosed area that limited ambient light. The touchscreens could be viewed through a 15 x 20 cm mesh window and could be reached through a 5 cm diameter arm hole that was surrounded by an antenna that read RFID chips implanted in monkeys’ arms. Correct responses to cognitive tasks were reinforced with sucrose and nutritionally-balanced fruit-flavored pellets (93 and 96 mg respectively).

RFID microchips (12 mm, Biomark, Boise, ID) were implanted in both forearms by subdermal injection for automated individual identification. Chips were implanted in all animals in the group in April, 2010, while animals were anesthetized for routine veterinary access. Animals born into the group after that date received their RFID chips at their first routine veterinary check, generally within their first year of life. The RFID reader at each testing station was connected to a computer that controlled stimulus presentation and recorded data. When a monkey put an arm through the arm hole to touch the touchscreen, the reader identified the monkey, the computer selected the appropriate task and trial for that monkey, and responses were recorded in a monkey-specific data file. The four testing units were connected to a central server that allowed for coordination, such that an animal could seamlessly move between testing stations without missing a trial or losing data.

The automated cognitive testing systems were available 24 hours per day, 7 days per week. Data were collected from April 9th 2010 through March 31st 2014.

### Cognitive tasks

Training began with a shaping procedure. In the first phase a randomly-chosen clip art image was displayed in a pseudo-random location on the touchscreen, biased toward the most easily reached locations on the screen. Animals received a reward for touching anywhere on the screen. Once monkeys completed 100 trials, they moved to phase two in which they were required to touch within the image boundaries (FR1) to receive a reward. After 100 trials on phase two, they moved to phase three in which they touched a green start square in the lower center of the screen (FR2) followed by an image (FR2). They were considered to have passed shaping training after 100 successful trials of stage 3. Latency to pass training was calculated as the difference between the date on which each animal received RFID chips and the date on which it completed Phase 3.

Animals that completed initial training participated in different cognitive tests over the course of the study. Each animal progressed independently, moving on to new cognitive tasks as soon as they completed training. As a result, different animals were engaged in different stages of training and different cognitive tasks at the same time. Allowing such ad lib access to the cognitive testing equipment and self-paced testing was a major goal of this work. Experiments included matching-to-sample, perceptual discrimination, perceptual classification, transitive inference [15], and two social categorization tasks. On average, monkeys that passed initial training participated in 2.44 ± 0.23 tasks over the course of the study. More information about participation in the cognitive tasks is provided in S1 Table. Cognitive task type and accuracy were not included as variables in the following analyses, as previously published results from this cognitive testing system suggest that there are no systematic differences in performance based on rank or sex [15], and assignment to cognitive tasks was not made based on any of the demographic factors analyzed in the present study, such that cognitive task is not confounded with any demographic variables.

## Study 1: Factors related to completion of initial touchscreen training

### Methods

#### Statistics

All statistical analyses were conducted in R version 3.4.0 [46] using a p value of .05. Where appropriate, means are listed as M + SD.

#### Social rank

The dominance hierarchy of the group was determined through creation of a matrix of dyadic interactions [47, 48] based on *ad libitum* observation of dominant and submissive behaviors (displacement, threat, chase, attack, and fear grimace) gathered at the beginning of the study (May 2010 to August 2010). As rhesus monkeys are a matrilineal species where daughters inherit their mother’s rank [49], juveniles were assigned to the same rank as their mothers. The behavioral management team at the Yerkes National Primate Research Center where the animals were housed conducted weekly observation of the group to determine whether any major changes in rank structure occurred. Based on their observations and personal observations by RPG, there were no major changes to the social structure of the group during the study period. A major change to the group hierarchy did occur in April 2014, when the highest ranking matriline was overthrown by a lower ranking matriline. As a consequence of this rank overthrow, the study period for the present analyses ended prior to this change, in March 2014.

The 30 adult females and three adult males present in the group in May of 2010 were divided into high, medium, and low rank categories of 11 animals each [15]. These categorical groupings allowed for consistent and sufficiently large subject numbers in each rank category to perform the rank comparisons of touchscreen activity presented in Study 2. The total number of monkeys (adults and juveniles) and total number of females in each rank category are provided in Table 1. To determine if there was a more nuanced difference between ranks, models were rerun using matriline rank order as a continuous variable where statistically possible. As in the categorical analysis, juveniles were assigned the same rank as their mother.

**Table 1:** General group demographics and touchscreen use measures across the three rank categories.

	Total	Low Ranking	Medium Ranking	High ranking	Statistical comparison of rank categories
Total number of animals (% of group)	128	46 (35.9%)	40 (31.2%)	42 (32.8%)	χ^2^_2_ = .438 *p* = .804
Number of females (%)	88 (68.8%)	29 (63.0%)	31 (77.5%)	28 (66.7%)	χ^2^_2_ = .159 *p* = .924
Age at training in months (Mean ± SD)	40.44 ± 43.65	40.77 ± 38.02	43.02 ± 45.75	37.79 ± 46.95	ANOVA F_2, 125_ = 0.16 *p* = .855
Latency to pass training in months (Mean ± SD)	7.68 ± 6.78	10.55 ± 9.16	6.28 ± 5.50	6.20 ± 3.73	ANOVA F_2, 54_ = 2.61 *p* = .083
Number of animals passing training (%)	57 (44.5%)	18 (39.1%)	26 (65.0%)	13 (31.0%)	See section 1.

#### Factors related to completion of initial touchscreen training

To determine the relations of demographic factors with *whether* monkeys completed training, monkeys were coded as either having completed all three phases of touchscreen training (1) or not (0). A logistic regression model (gam function) [50] was used to analyze the relation of three static factors (sex, age at training, and rank; detailed results presented in S1 Table). Given that the average age of males was lower than that of females, the interaction of sex and age at training was also included in the model. Logistic regression is similar to linear regression but can be used with binary data (did the monkey complete training or not?) [51]. Importantly, logistic regression models estimate odds ratios, which are functions of proportions. For each group in a comparison (for example, males vs females) a proportion is calculated by dividing the number of individuals who completed training by the number of individuals in that group. These proportions allow for unbiased comparison of the probability that monkeys complete training, while accounting for differences in the number of monkeys in the groups. All results presented in the results section below come from this single logistic regression model unless otherwise specified. A follow up independent t-test was performed to clarify sex effects. Statistical models were fit using the nlme [52] package for R.

### Results and discussion

#### Group demographics

Chi squared goodness of fit and analysis of variance tests revealed that the three rank groups (high, medium, and low) did not differ in the total number of animals, number of females, age at training, and latency to pass training (Table 1).

Independent t-tests revealed that the average age at training was lower for males than for females (males = 19.50 ± 19.57 months; females = 49.95 ± 48.13; t_126_ = 3.85, *p* < .001), and that females had significantly more months of access to the touchscreen systems than did males (mean duration in group during study period: males = 29.53 ± 14.85 months; females = 37.87 ± 8.54 months; *t*_126_ = -4.02, *p* < .001). This is likely due to the colony management practice of removing males at puberty. Overall, there were more females present in the group (Table 1), females were older when training, and females had more time over which to interact with the cognitive testing systems. These differences were taken into account in our statistical analyses through inclusion of sex and age factors in the analyses (see below).

#### Factors related to completion of initial touchscreen training

**Sex and age.** The logistic regression revealed that females were more likely to complete training than males (*z* = 2.21, *p* = .027; detailed results presented in S2 Table). This finding is not just due to the higher number of females in the group compared to males (Table 1), as logistic regression accounts for differences in the number of monkeys of each sex. This finding could be due to the increased time females spent in-group compared to male, as this additional time created more opportunities to complete initial shaping training. However, a follow up independent samples t-test revealed that males and females did not differ in how long it took them to complete initial training (females = 7.26 ± 6.69 months, males = 8.44 ± 6.87 months; independent samples t-test: *t*_55_ = 0.55, *p* = .642), indicating that having more months of access to the touchscreens did not impact the likelihood that females completed training. Both males and females tended to either engage with the system and complete training shortly after receiving their RFID chip or did not ever do so (S3 Fig). Additionally, 83% (33/40) of males were present in the group after receiving their RFID chips for at least the 8.44 months that the average male required to complete initial training (M = 29.52 ± 14.85 months). Relatively low levels of participation by males was therefore unlikely due to less time in group, as most males were present in the group for a sufficient amount of time to have completed initial training.

The trend for females to be more likely to complete training could also have been driven by age, because females were older on average when they received access to the system. However, the logistic regression revealed the opposite effect of age at training (z = -3.17, *p =* .002), such that the older monkeys were when they received access to the touchscreen systems, the less likely they were to have passed training. Additionally, there was no significant interaction between sex and age at training on training completion (*p* = .915), indicating that the effect of sex was not driven by age differences.

That females were more likely to complete training than males, and thereby increase their food intake through cognitive testing rewards, may be consistent with findings that wild female monkeys spend more time foraging than do males [30–32, 53]. This sex difference could be due to many other causes, including increased interest in the testing equipment, fewer competing activities, or differences in social influences. For example, if monkeys were selectively attracted to interacting with the touchscreen system at the same time as same sex individuals, this would lead to higher activity from females due to the higher number of preferred testing partners (see Study 2 for analysis).

#### Social rank

In contrast to predictions based on ecology and prior reports, the logistic regression model did not reveal a difference in completion of training between low and high or low and medium ranking monkeys (Table 1; low vs. high: *p* = .113; low vs. medium: *p =* .101). Interestingly, medium ranking monkeys were more likely to have completed training than were high ranking monkeys (medium vs. high: *p* = .002). A follow up analysis that reran the same model using rank as a continuous variable also found no effect of rank (*p* = .207). Importantly, these patterns indicate that high-ranking animals did not dominate the testing equipment to the exclusion of low and medium ranking animals. Monkeys of all ranks therefore had sufficient access to the touchscreen systems to complete initial training. However, there was a trend (Table 1) for lower ranking monkeys to take longer to complete training. It is possible that lower ranking monkeys may learn more slowly than higher ranking monkeys. Alternatively, this pattern may suggest that access to the cognitive testing systems was more constrained for low ranking monkeys than for high and medium ranking monkeys (see Study 2 for analysis).

#### Summary

Overall, females and animals who were younger when they first received access to the testing system were more likely to have completed training than males. This is consistent with previous findings that younger animals were more likely to participate in cognitive testing in wild great tits and captive baboons [17, 23]. However these results contrast with previous findings that sex was not related to cognitive test participation in great tits [23], and that socially-housed male baboons interacted more with an automated touchscreen system than females [13]. Additionally, in contrast to findings in baboons, in which 75% of eligible monkeys participated in testing [54], only 45% of rhesus monkeys passed initial training. These differences in the relations of demographic factors to participation in cognitive testing across testing systems and species suggest that differences in social structure, group dynamics, sex ratios, feeding schedules, or details of the testing systems may result in different patterns of participation.

## Study 2: Factors related to amount and patterns of touchscreen activity

While the first study focused on how demographic factors relate to *whether* a monkey interacted with the touchscreen system, and thus whether they completed the short initial training, other factors may be related to *how much*, *when*, and *with whom* individual monkeys interact with the system. Monkeys who completed training were presented with a variety of cognitive tasks to complete at their own pace, and as there were four adjacent testing systems, multiple monkeys could participate in cognitive testing at the same time. We determined the extent to which sex, age at training, age at testing, reproductive experience, and social rank related to 1) amount of touchscreen activity, 2) daily trends in touchscreen use, and 3) which pairs of monkeys worked concurrently on the touchscreen systems.

### Methods

All analyses in this study use only data from the 57 animals who completed training.

#### 1. Factors related to amount of touchscreen activity

Engagement with the touchscreen system was measured as the total duration of trials completed (in seconds) for each subject in each full month they were present in the group. Trial duration was the best measure of touch screen engagement because it captured the total amount of time each animal devoted to cognitive testing, and therefore controlled for any differences in the duration of trials in different tasks. The month by month analysis allowed us to control for the time each monkey spent in the group, as a monkey would not be included in the data set for a month in which it was not present. Time spent testing was log transformed (LOG[duration + 1s]) to better meet normality assumptions.

A generalized additive mixed model [55] was used to determine the effect of fixed factors sex, age at training, age at testing, rank, and a smoothed effect of time of year on the time monkeys engaged in testing each month (detailed results presented in S3 Table). Generalized additive mixed models are similar to linear regression except they allow for repeated measurements from each subject (e.g. data for each subject for each month they were present in the group) and for data patterns that are curvilinear (e.g. variation across seasons). The first three factors (age at training, sex, rank) were statically-coded for each individual and did not change over the course of the experiment. Given that the average age of males was lower than that of females, the interaction of sex and age at testing was also included in the model. The final two factors (age at testing, time of year) changed dynamically across trials, and were therefore coded trial by trial. Age at testing was defined as the monkey’s age in months when a given trial was run. Time of year was coded as the month in which a trial was initiated. Time of year smoothing was done using cyclical cubic splines which ensured that seasonal estimates met at the start and end of the year [51]. The model additionally included a random subject-specific intercept to account for the lack of independence due to repeated measures from individual monkeys [55, 56]. Models were fit using the mgcv package [50] for R.

#### 2. Daily trends in touchscreen use

To determine how high, medium, and low ranking monkeys distributed their work across the day, we examined whether rank and time of day were related to the amount of touchscreen activity. The time of a trial was defined as the start time for each trial. Time was grouped into six three-hour blocks (6:00–9:00, 9:00–12:00, 12:00–15:00, 15:00–18:00, 18:00–21:00, 21:00–24:00). No touchscreen activity occurred between 24:00–6:00, therefore these times were not included in the analysis. The total time spent interacting with the touchscreen during each of the six time blocks in each month of testing was calculated for all 57 animals who passed training.

A generalized additive mixed model was applied to the log transform of seconds spent interacting with the touchscreen system in each time block (+ 1 second). The model contained fixed factors of sex and age at training with a subject-specific random intercept. A smoothed effect of time of day was fit by rank category (low, medium, high), resulting in a smoothed curve for each of the three rank categories. Pairwise comparisons of these curves between the rank categories (i.e. low vs. high, high vs. medium, and low vs. medium) were conducted using a G statistic derived using the methods of Zhang and Lin [57] in MATLAB [58]. A p value < .05 on these pairwise comparisons indicates a significant difference between the smoothed curves (detailed results presented in S6 Table). Models were fit using the mgcv package [50] for R.

#### 3. Factors related to which monkeys work concurrently

Data on the start and end time of every trial completed by each monkey over the course of the study were mined using MATLAB to generate a matrix of the total number of seconds that each pair of monkeys overlapped on the touchscreens. The computers only recorded trial starts and ends, therefore times when a monkey was sitting at a computer but not actively completing a trial were not recorded and were not used to calculate overlap. We used a valued exponential random graph model (ERGM) [59, 60] to determine the extent to which being a specific rank (node factor of rank), sharing a rank (node match of rank), sharing a matriline (node match of matriline), and being of the same sex (node match of sex) affected the total time for which dyads overlapped on the touchscreens (for further details see S1 File). Importantly, differences in the number of individuals in each demographic group (sex, rank) are taken into account by the model when calculating the probability of an overlap [61]. The sum of the edge weights was included as a structural covariate, similar to including an intercept in a linear model. Lastly, to control for differences in possible overlap time across the dyads (i.e. how many months two monkeys overlapped in the group based on date at RFID chipping and removal from the group), the log number of months (plus a small factor of 0.01 months) in which a dyad could have overlapped on the touchscreen system was added as an edge covariate term [62]. The Poisson-distributed response for the ERGM was the total number of seconds of overlap over the course of the study for the two individuals. The model was run on a computing cluster using the ergm package for R [63] (detailed results presented in S7 Table). Diagnostics for the MCMC chains and goodness of fit plots for the model are available in S1 File.

### Results and discussion

A total of 1,652,738 cognitive testing trials were completed over the course of the study. Monkeys spent 4.92% of the time they had access to the touch screens (6:00–24:00) interacting with the touchscreens. The touchscreen systems were therefore unoccupied over 95% of the time, indicating that there was not intense competition for access to the systems.

#### 1. Factors related to amount of touchscreen activity

**Sex and age.** Consistent with our finding that females were more likely to complete training, females who had completed training also interacted with the touchscreens significantly more per month than males who had completed training (b = 3.39, SE = 1.54, t_1_ = 2.21, *p* = .028). Neither age at training (*p* = .128) nor age at the time of testing (*p* = .666) were predictive of the amount of monthly interaction with the touchscreen systems, and the interaction between sex and age at testing was not significant (*p* = .507). This indicates that the sex effect is not driven by differences in age distribution between males and females. Correct responses to the cognitive tests resulted in food rewards, mimicking natural foraging. Therefore interaction with touchscreen systems may be governed by the same factors that result in higher rates of foraging in female primates [11].

**Social rank.** Consistent with our findings on training completion rates, there were no significant differences in monthly activity between high and low ranking monkeys (b = 1.64, SE = 1.11, *t*_1_ = 1.48, *p* = .139) or between medium and high ranking monkeys (b = 0.30, SE = 1.03, *t*_1_ = 0.29, *p* = .772). However medium ranking monkeys did use the touchscreens significantly more than low ranking monkeys (b = 1.93, SE = 0.93, *t* = 2.09, *p* = .037). A follow up analysis that reran the model with rank as a continuous variable did not find an effect of rank (*p =* .116). The lack of a dramatic difference in touchscreen use between the high and low ranking monkeys indicates that individuals had regular access to the touchscreen systems regardless of rank. Competition for the testing systems may have been ameliorated by having four systems available, allowing multiple animals to participate in testing at the same time, rather than competing for access to a single unit. Importantly, that high ranking monkeys did not dominate the testing systems even in highly despotic rhesus monkeys [64] provides further evidence for the viability of automated cognitive testing systems in animals housed in naturalistic social groups.

**Fig 2:**
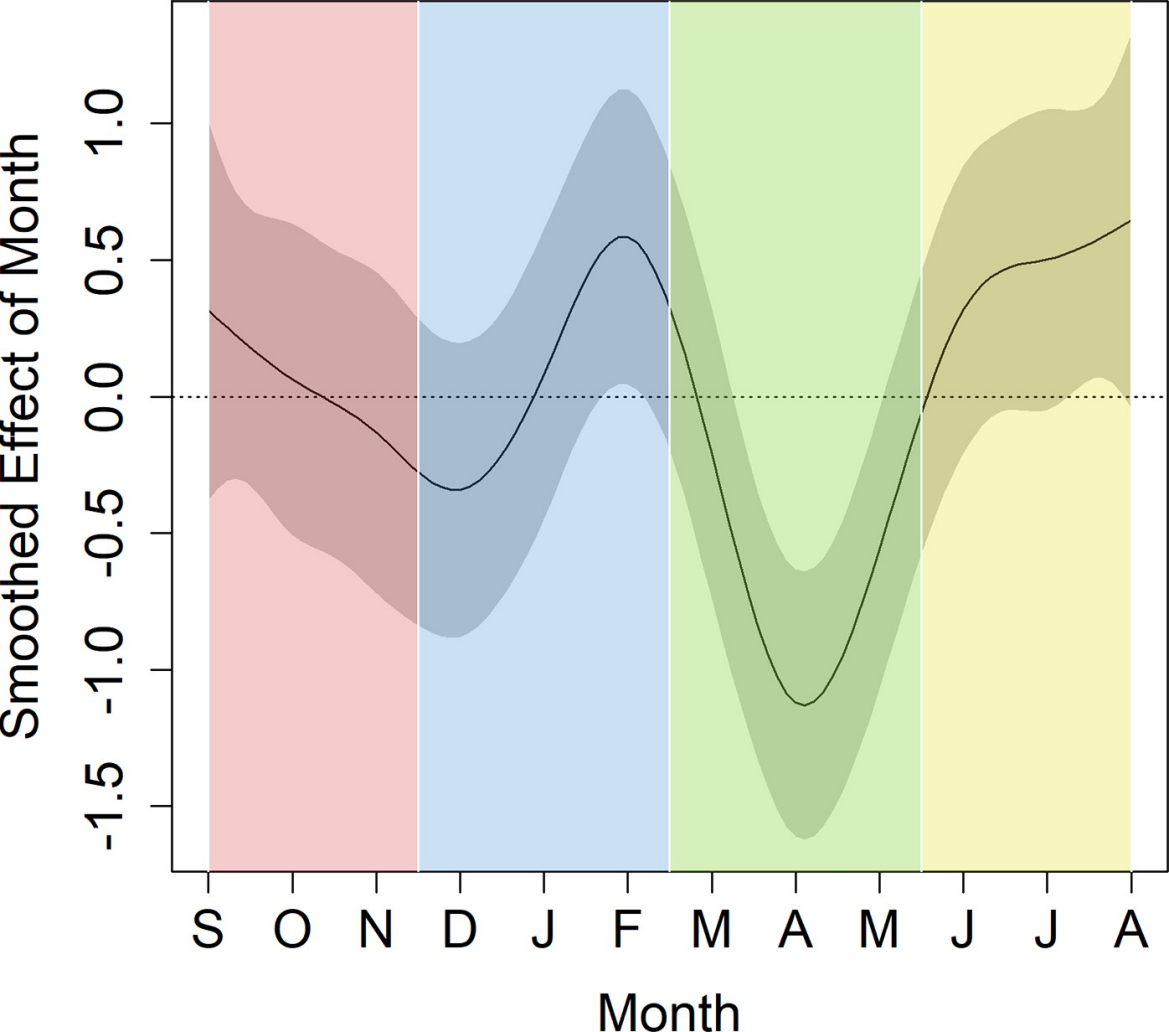
Smoothed effect of month on the amount of touchscreen activity for all females. Gray bands represent 95% confidence intervals for the fitted smooth effect. Areas where the gray bands do not overlap with the central y = 0 line reflect areas of significant seasonal effect. The x-axis is scaled in months, beginning in September and ending in August. The colored vertical bands represent the different seasons.

**Seasonality and reproductive experience.** Engagement with the touchscreen system varied seasonally (F_6, 6_ = 3.481, *p* < .001). Mirroring the cycle of mating in the fall and birthing in the spring, there was a decrease in touchscreen activity during the birthing season (March to May; Fig 2) and peaks in activity during the non-breeding seasons (winter [February] and summer [July]). This is consistent with findings that vervet monkeys spend significantly less time foraging during the birthing season than other times of the year [40]. For the vervets, this may be driven by seasonal changes in food availability, as the food available in the non-breeding season takes longer to process than the food available in the birthing season. Alternatively, the vervet feeding pattern could be driven by changes in individual activity budgets due to the addition of the demands of parental care, as females spend more time in infant care activities in the birthing season [40]. Similarly, our observed seasonal changes in touchscreen activity could be driven by general group-level seasonal shifts, such as differences in the weather, or to individual changes in activity budgets for breeding-aged females caused by the addition of mating and infant care activities to daily activity budgets. Importantly, because the animals were maintained in captivity, the availability of food was constant across the seasons.

To investigate the cause of the observed seasonal variation in testing activity, we re-ran the model on two distinct subsets of data: one on breeding-aged females (3 years or older, i.e. animals who would be engaged in mating and/or birthing; detailed results presented in S4 Table) and one on non-breeding-aged females (less than 3 years of age, i.e. females that would not be engaged in mating and/or birthing; detailed results presented in S5 Table). Due to the small number of males who completed initial training we did not have sufficient data to run this follow-up analysis on males. The model run for non-breeding aged females was identical to the original, while the model for breeding-aged females included one additional fixed factor, reproductive status (0 = nulliparous, 1 = parous). Parity changed from 0 to 1 over the course of the experiment for those females who gave birth to their first infant during the study period. Females who turned three during the study period moved into the breeding-aged analysis in the month following their birthday.

If seasonal changes in touchscreen activity were driven by changes such as weather that affect all the monkeys, both breeding and non-breeding aged females should show a seasonal activity curve consistent with the breeding cycle. However, if use of the touchscreen computers varied seasonally due to individual-level changes in activity budgets for breeding-aged females during the mating and birthing seasons, breeding-aged females should show seasonal variation consistent with this cycle (decreased activity in the breeding seasons) while non-breeding aged females should show no seasonal variation. Further, there should be a significant effect of reproductive experience, such that females who are parous, and therefore actively engaged in infant care, should show decreased touchscreen use compared to non-parous breeding aged females.

**Fig 3:**
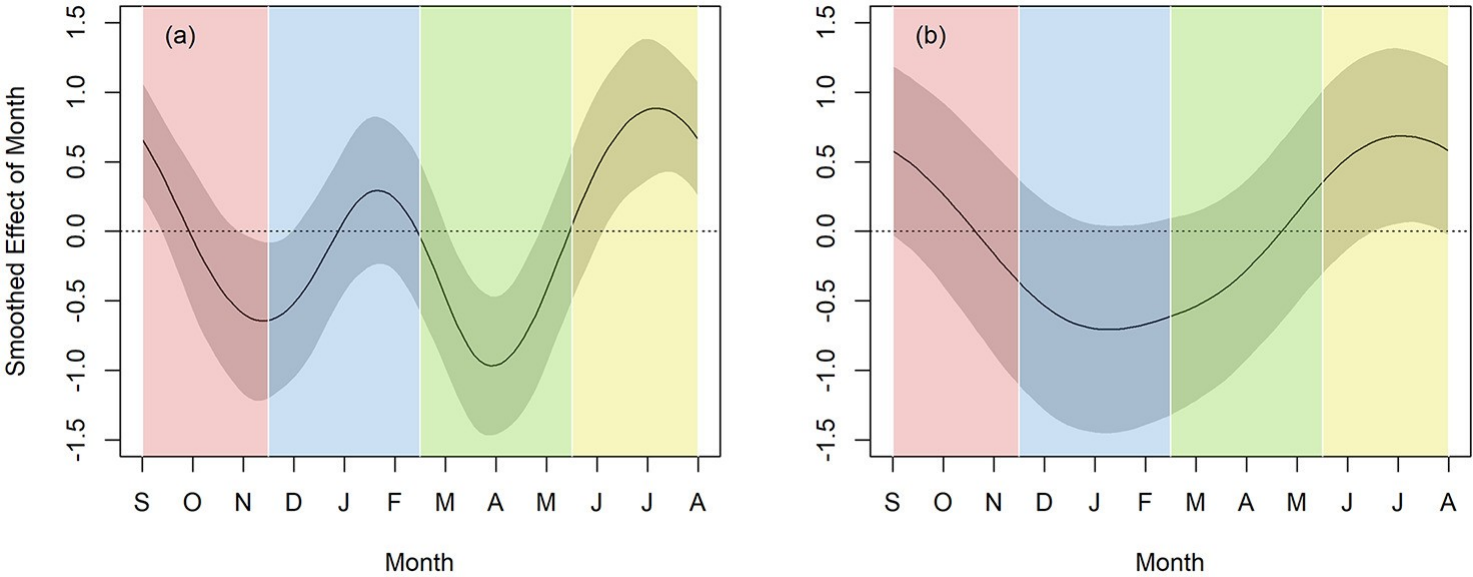
*Left*. Amount of touchscreen activity by season for females of breeding age (> 3 years). *Right*. Amount of touchscreen activity by season for females of non-breeding age (< 3 years). Black lines represent means, grey areas represent 95% confidence intervals. When the grey line does not overlap with the central y = 0 line, the amount of touchscreen activity is significantly different from chance. The x axis is scaled in months, beginning in September and ending in August. The colored vertical bands represent the different seasons.

Both breeding and non-breeding-aged females showed a significant effect of seasonality (breeding-age females: F_4, 9_ = 3.27, *p* < .001, Fig 3 *left*; non-breeding-aged females: F_1, 9_ = .75, *p* = .018; Fig 3 *right*). However, the effect was different between the two groups, such that breeding-aged females showed a pattern consistent with the breeding cycle while non-breeding aged females did not. Breeding-aged females showed decreased activity during the mating season (November) and birthing season (March to May) and increased activity in the summer (June to August). In contrast, non-breeding-aged females demonstrated a stable pattern of working through most the year, with a small spike in activity at the summer (July). For breeding-aged females, reproductive experience also had a significant effect on touchscreen activity, with parous females engaging with the touchscreen system less than nulliparous females (b = -1.44, SE = 0.42, t_1_ = -3.46, p < .001). Together, these results suggest that females who were actively involved in mating, birthing, and infant care decreased cognitive testing seasonally and over their lifetime to accommodate these added activities.

For breeding-aged females, age at training and testing did not impact activity (all *p*’s > .05). However, for non-breeding-aged females both age at training and age at testing were related to activity on the touchscreen systems. The younger non-breeding aged females were when they started training, the more they participated in testing (b = -0.22, SE = 0.06, t_1_ = -3.42, p < .001). Additionally, non-breeding aged females worked more as they aged (b = 0.33, SE = 0.04, t_1_ = 8.47, p < .001). Rank was not significantly related to monthly touchscreen activity in either breeding or non-breeding aged females (*p*’s > .10).

**Summary.** Overall, our results indicate that socially-housed female rhesus monkeys participate in cognitive testing more than do males. For these females, juveniles steadily increase their interaction with the touchscreen devices until they reach adulthood, at which point age is no longer a factor in touchscreen activity. Instead, touchscreen activity for adult females is modulated by mating and birthing, such that females work less once they have bred and show decreased touchscreen activity during both the mating and birthing seasons. Consistent with females seeking out additional calories while nursing, adult females show increased touchscreen activity in summer. These patterns of activity are consistent with literature suggesting that female monkeys alter feeding and foraging behavior during breeding seasons [40, 65, 66], and suggests that participation in cognitive testing is subject to similar seasonal pressures as are natural behaviors.

#### 2. Daily trends in touchscreen use

**Fig 4:**
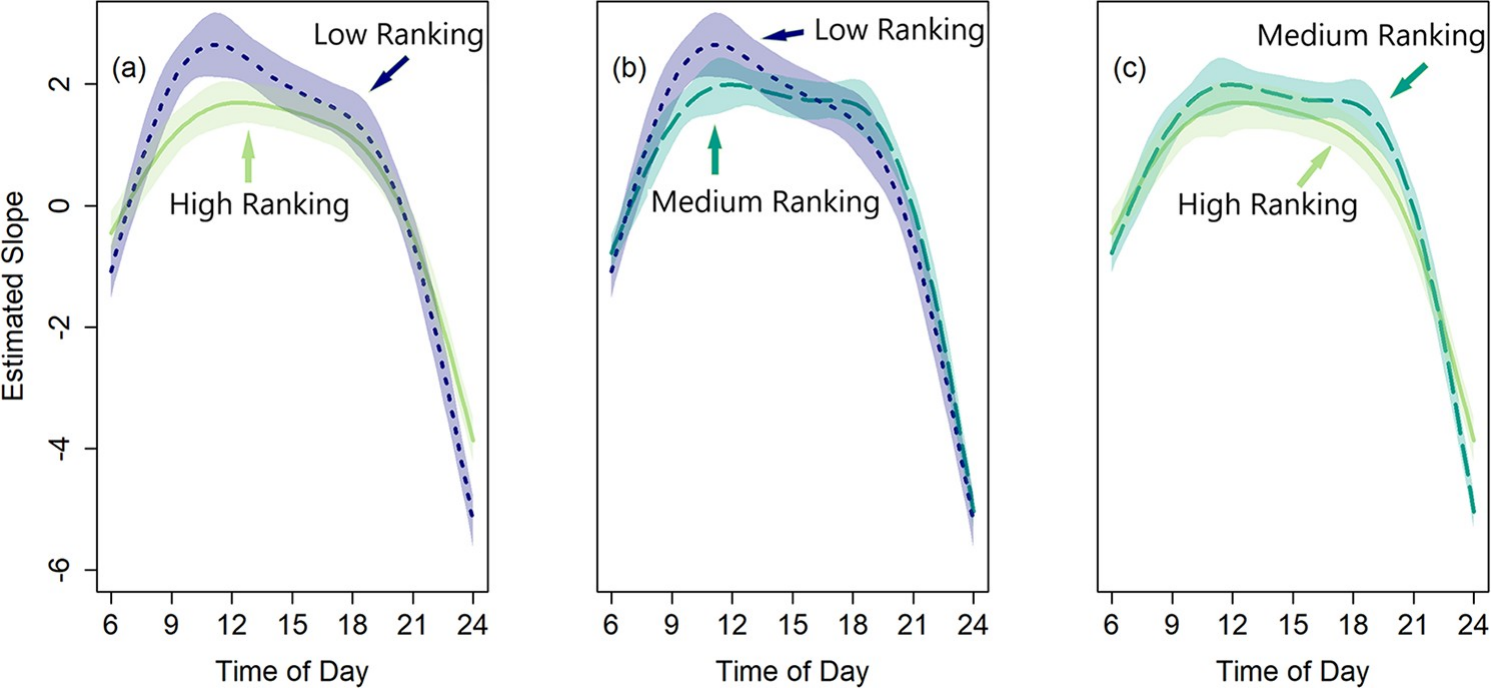
Amount of touch screen activity relative to baseline over the course of a day. Each time block is a period of three hours. Lines represent fitted means, shaded areas represent 95% confidence intervals. Areas where the shaded regions do not overlap are areas of significant difference between the two curves.

While the rank groups did not differ in their likelihood of completing training or in their amount of interaction with the touchscreen system, they may show different patterns of activity that minimize conflict similar to those seen in natural feeding behavior. Indeed, there were significant differences between all rank categories in how they distributed their touchscreen activity throughout the day (Fig 4; high vs low: χ^2^_7_ = 104.84, *p* < .001; high vs medium: χ^2^_4_ = 117.25, *p* < .001; medium vs low: χ^2^_1_ = 141.65, p < .001). Specifically, the low ranking monkeys worked more than the high ranking monkeys between 9am and 12pm (Fig 4). There were also brief periods of difference between the high and medium ranking monkeys (after 11pm) and between the medium and low ranking monkeys (around 10 am). These differences in how the three rank categories distributed their touchscreen activity support the idea that monkeys distribute their time on the systems to minimize conflict. There was a significant effect of time of day on activity, with high, medium, and low ranking monkeys each showing a significant effect of time block (all *p*’s < .001), indicating changes in activity across the day. Sex (*p* = .289) and age at training (*p* = .283) were not related to the amount of touchscreen testing across the six time blocks for an individual.

High ranking monkeys show a reasonably flat distribution across daylight hours, suggesting that they engage with the touchscreen system without constraint. In contrast, low-ranking monkeys showed a spike in activity in the morning followed by a sharp decline over the rest of the day. This suggests that low-ranking monkeys may capitalize on the fact that high and medium ranking monkeys are engaged in foraging activity in the late morning to engage with the touchscreen systems in a way that minimizes the opportunity for conflict. This finding that monkeys of different ranks interact with the touchscreen systems at different times is consistent with findings that low and high ranking monkeys feed at separate times, with the high ranking monkeys generally approaching food first, and the low ranking monkeys approaching to feed after a delay [36, 67, 68]. That patterns of touchscreen use appear similar to patterns of feeding with animals distributing testing to avoid conflict between ranks, suggests that even in despotic species like rhesus monkeys, automated testing systems can be used to collect data from animals of all ranks with no interference required from experimenters.

#### 3. Factors related to which monkeys work concurrently

**Fig 5:**
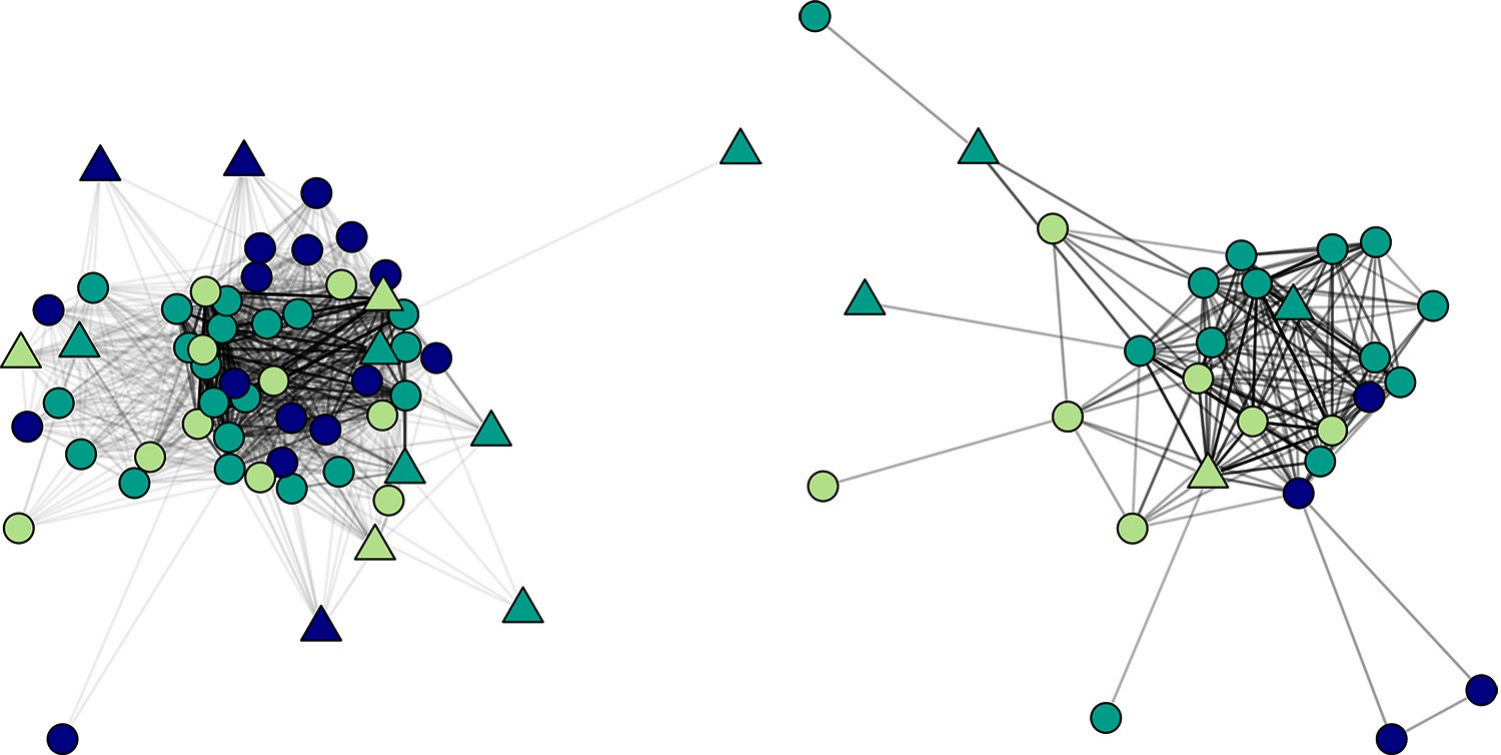
Social network of overlap on touchscreen systems. Edges (lines) represent two animals working together on the touchscreen system. Darker lines represent more monthly overlap than lighter lines. Males are represented as triangles and females are represented as circles. The color of the shapes reflects the animal’s rank grouping, where the light green is high ranking monkeys, dark green is medium ranking monkeys, and navy is the low-ranking monkeys. *Left*. Network for all individuals and all relationships. Low ranking monkeys tend to cluster together, while high and medium ranking monkeys are spread more widely. *Right*. Network when only the top 10% of relationships (in terms of strength) are plotted. Few low ranking monkeys appear on this graph, as they rarely show high levels of overlap with others.

The model converged after eight iterations for MCMLE. Interestingly, monkeys did not work more than expected by chance with individuals from their own matriline (node match of matriline: b = -1.10, SE = 1.21, *p* = .36]. However, consistent with findings from affiliative social behaviors such as play and grooming [69–71], monkeys worked more with individuals of the same sex (Fig 5; node match of sex: b = 3.20, SE = 0.66, *p* < .001). Importantly, ergm takes into account the number of possible pairs when calculating the probability of a node match, so this effect is not simply due to the higher number of possible female-female pairings. However, this result that monkeys are selectively attracted to working with same sex individuals may still partially explain the increased participation in cognitive testing by female monkeys overall. If monkeys are selectively attracted to interacting with the touchscreen systems when an animal of their sex is already interacting with the system, the increased number of females in the group would lead to more opportunities for concurrent activity for females than for males.

Consistent with time of day findings that suggested low ranking monkeys were avoiding co-working with high ranking animals, monkeys worked more with individuals from their rank category (node match of rank category: b = 0.77, SE = 0.38, p = .041) and low-ranking monkeys worked alone more frequently than did high-ranking monkeys (node factor of rank: low vs. high: b = -0.92., SE = 0.41, *p* = .024; low vs. medium: estimate = 0.23, SE = 0.38 *p* = .539; high vs. medium: estimate = -0.69, SE = 0.12, *p* = .067). This pattern can be seen in the social network graphs (Fig 5). When the network is thresholded to show only dyads that co-work often (i.e. the 90^th^ percentile; Fig 5 right), few low ranking monkeys appear on the network, indicating that they do not overlap with others as often as do high and medium ranking monkeys. These findings support the idea that low ranking monkeys minimized competition- in this case by preferentially interacting with the touchscreens when no other monkeys were present or only when low ranking animals are present. This tendency to avoid working with higher ranking monkeys may result in improved cognitive data, as low ranking monkeys have been shown to perform worse on cognitive tasks in the presence of higher ranking monkeys [27, 72].

## General discussion

Overall, about half of the monkeys present during the study completed initial training, qualifying them to participate in cognitive testing. Females were more likely to complete training than were males and interacted more with the touchscreen systems after initial training. Monkeys that were younger when they received access to the testing system were more likely to complete initial training. In females, age was positively related to touchscreen activity through adolescence, at which point seasonality and reproductive experience were stronger predictors of monthly touchscreen activity. This suggests that engagement with the touchscreen systems decreased when animals had additional demands on their activity budgets from mating, birthing, and infant care. Monkeys in different rank categories did not differ in how likely they were to interact with the touchscreen systems. However, low ranking monkeys appeared to time their use of the systems to minimize conflict by using the systems when they were not in use or were in use only by other low ranking monkeys.

Demographic data on automated touchscreen activity in naturalistic social groups has been published for two other species; Morand-Ferron’s operant testing system for wild birds [23] and Fagot’s ALDM for socially-housed captive Guinea baboons [13]. In wild great tits, 59% of banded birds participated in testing [23], sex was not a predictor of touchscreen activity [17, 23], and the effect of age varied by study, with one study showing no effect of age [23] and the other finding that juvenile birds completed more trials than adult birds [17]. Dominance rank data were not available. In a large group of socially-housed Guinea baboons, 75% of eligible monkeys participated in testing, males participated more than females, age was a positive predictor of touchscreen activity, and rank did not predict touchscreen activity [23]. This contrasts to findings from smaller social groups of Guinea baboons in which rank did impact testing: a high ranking female inhibited the ability of low ranking females to interact with the testing system, resulting in little or no data from the low ranking monkeys [14]. Importantly, in the case where no effect of rank was found, 10 cognitive testing units were available in the group, while in the case where rank did impact access, only one cognitive testing unit was available. This, combined with our findings that rank did not impact touch screen activity when four testing units were available, suggests that in primates, providing multiple systems may decrease competition and decrease the ability and interest of high ranking animals to monopolize the testing systems. Methodological details such as the number of testing stations and differences in species behavior or ecology may have important impacts on how many, and which, animals participate in testing. For example, differences in feeding schedules, species social organization, and availability of privacy during testing may explain the comparatively low participation rate in our study compared to that show by baboons [13]. This highlights the importance of understanding the ecological and social factors specific to the test species when designing automated cognitive testing systems [23].

Overall, participation in cognitive testing followed patterns similar to those shown by primates engaged in natural foraging [11]. Females participated more than males [30, 32, 33], participation varied across seasons [31, 40, 65, 66], and low ranking monkeys obtained access by avoiding higher ranking monkeys [36, 67, 68]. This supports research from socially-housed captive chimpanzees in which providing foraging opportunities in the form of cognitive testing resulted in activity budgets more similar to wild than captive chimpanzees [11]. Baboons preferentially participate in touchscreen computer testing near social partners with whom they tend to forage [44], suggesting that even social overlap in cognitive testing may mirror foraging patterns.

On cognitive tasks such as those in the present study, monkeys are reinforced for correct choices but not for incorrect choices. Demographic patterns of cognitive testing participation could therefore be mediated by differences in sensitivity to this reinforcement. For example, testosterone, estrogen, and progesterone have been shown to moderate sensitivity to reinforcement, with increased sensitivity associated with increased testosterone and estrogen levels [73, 74]. Males, or females at their estrogen peak, may therefore be more motivated to participate in cognitive testing. However, that females participated in cognitive testing more than males, and that cognitive testing participation was at its low for females during the period when their estrogen levels were at peak (breeding season), do not suggest such effects in our data set.

There has been a recent surge in the number of studies attempting to clarify the relations between ecology, behavior, and cognition [4, 75]. These questions are essential to our understanding of the evolution of cognition and of how cognitive processes function in natural social environments [4]. Due to the difficulty in obtaining repeated samples from large numbers of subjects and to the testing methods available for field experiments, studies of cognition in the wild generally cannot elucidate the mechanisms underlying performance [75]. Automated cognitive testing systems in naturalistic social groups may provide at least a partial solution to these concerns, as they allow for repeated testing of identified individuals using testing methods similar to those traditionally employed in the lab.

Comparisons of cognitive performance by naturalistically and laboratory-housed birds and primates suggest that automated cognitive testing systems result in performance similar to that seen in the lab [15, 23] and that animals housed in naturalistic social groups will participate even in demanding complex cognitive tasks [15, 76]. Importantly, while the results of the present study are based on a large number of subjects, they come from only one group. These results may therefore not apply to all groups of rhesus macaques. However, the present analyses indicate that automated cognitive testing can be an effective method for collecting large amounts of cognitive data on a range of cognitive tasks in a naturalistic social group of monkeys.

Given the demographic trends in participation, this method may be more difficult to use to address research questions focused on sex differences (few males participated in cognitive testing), effects of reproductive experience on cognition (parous females showed diminished activity), or seasonal variation in cognition (participation by adult females varied across the breeding cycle) in this species. Importantly, this method does provide sufficient data to conduct controlled tests of possible relations between rank, age, and cognition. While some studies may simply not be possible given colony management practices such as removing most adult males from the group at puberty, other imbalances in the availability of data, such as those related to season, may be ameliorated if monkeys receive all of their food from cognitive testing. Whether requiring cognitive testing of all animals in a colony is viable remains to be determined, and such systems would need to be developed with care to ensure the welfare of all subjects. Employing automated cognitive testing systems in the wild and in naturalistic captive groups could allow for the combination of behavioral, ecological, and rigorous cognitive data necessary to address complex questions about the evolution and function of cognition.

## Supporting information

S1 Fig MCMC diagnostics for Valued-ERGM. Left column shows trace plots of MCMC chains. The best plots show random noise around a mean value, indicative of convergence. The right plot shows a histogram of the parameter estimates. The results are broken down term-by-term. Rows show the results for the (a) sum term, (b) node match of sex, (c) node match of matriline, (d) node match of rank, (e) node factor of low rank, (f) node factor of medium rank, and (g) edge covariate of the log of the number of months of overlap.
(TIF)

S2 Fig Goodness of fit plots for Valued-ERGM model. Each panel shows a histogram of the distribution of statistics for n = 1000 simulated models from the observed model. The vertical bar shows the observed statistic from the data. The best fit models have observed statistics near the center of the distribution of simulated statistics. Panels show the distributions for the terms (a) sum, (b) node match of sex, (c) node match of matriline, (d) node match of rank, (e) node factor of low rank, (f) node factor of medium rank, and (g) edge covariate of the log of the number of months of overlap.
(TIF)

S3 Fig Histograms of latency to complete initial training. Females (*top)* and males (*bottom*). Dotted lines indicates mean latency.
(TIF)

S1 Table Information about cognitive tasks. All data present means ± standard deviations.
(PDF)

S2 Table Logistic regression results for analysis of factors related to completion of initial touchscreen training. (PDF)

S3 Table Generalized additive mixed model results for analysis of factors related to amount of touchscreen activity. (PDF)

S4 Table Generalized additive mixed model results for analysis of factors related to amount of touchscreen activity for adult females. (PDF)

S5 Table Generalized additive mixed model results for analysis of factors related to amount of touchscreen activity for juvenile females. (PDF)

S6 Table Generalized additive mixed model results for analysis of daily trends in touchscreen use. (PDF)

S7 Table Exponential random graph model results for factors related to which monkeys work concurrently. (PDF)

S1 File Social network description and diagnostics. (PDF)
