# Supplementary figures and images for: Influences of demographic, seasonal, and social factors on automated touchscreen computer use by rhesus monkeys (Macaca mulatta) in a large naturalistic group
Source: PLoS One. 2019 Apr 24;14(4):e0215060. doi: 10.1371/journal.pone.0215060 (PMC6481812; doi:10.1371/journal.pone.0215060)

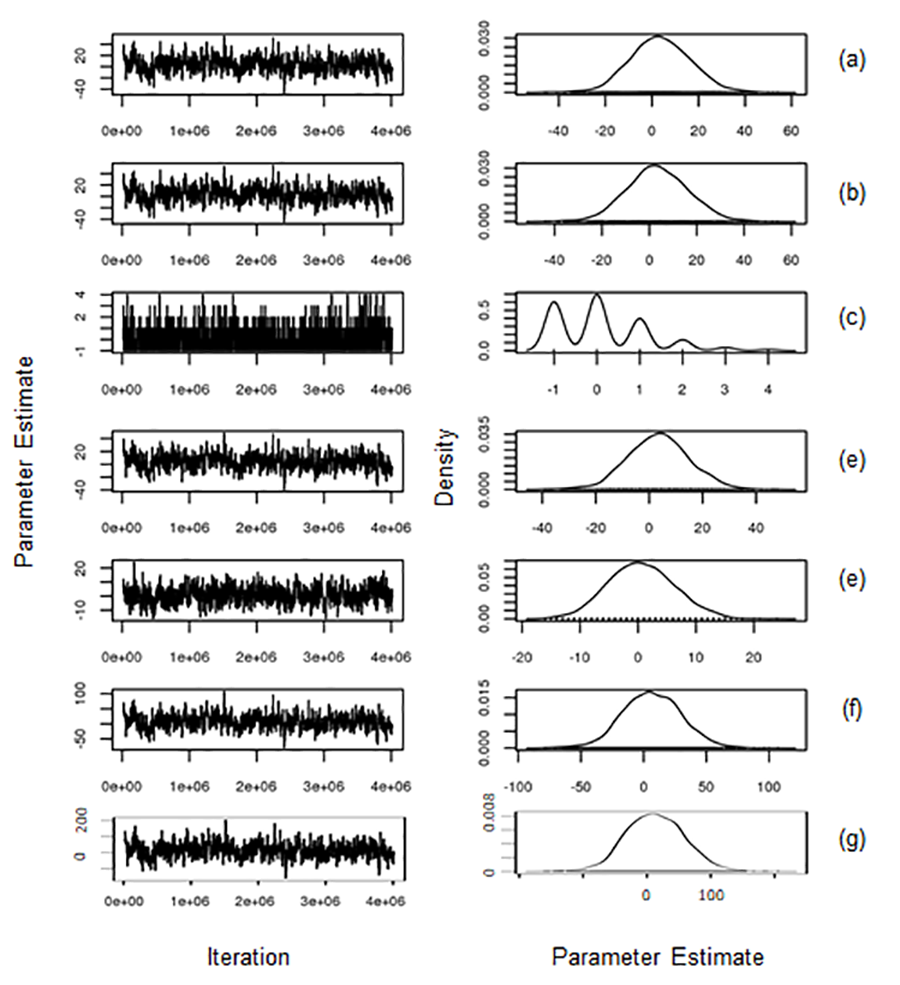

Supplement: S1 Fig — Left column shows trace plots of MCMC chains. The best plots show random noise around a mean value, indicative of convergence. The right plot shows a histogram of the parameter estimates. The results are broken down term-by-term. Rows show the results for the (a) sum term, (b) node match of sex, (c) node match of matriline, (d) node match of rank, (e) node factor of low rank, (f) node factor of medium rank, and (g) edge covariate of the log of the number of months of overlap. (TIF) [file pone.0215060.s001.tif]

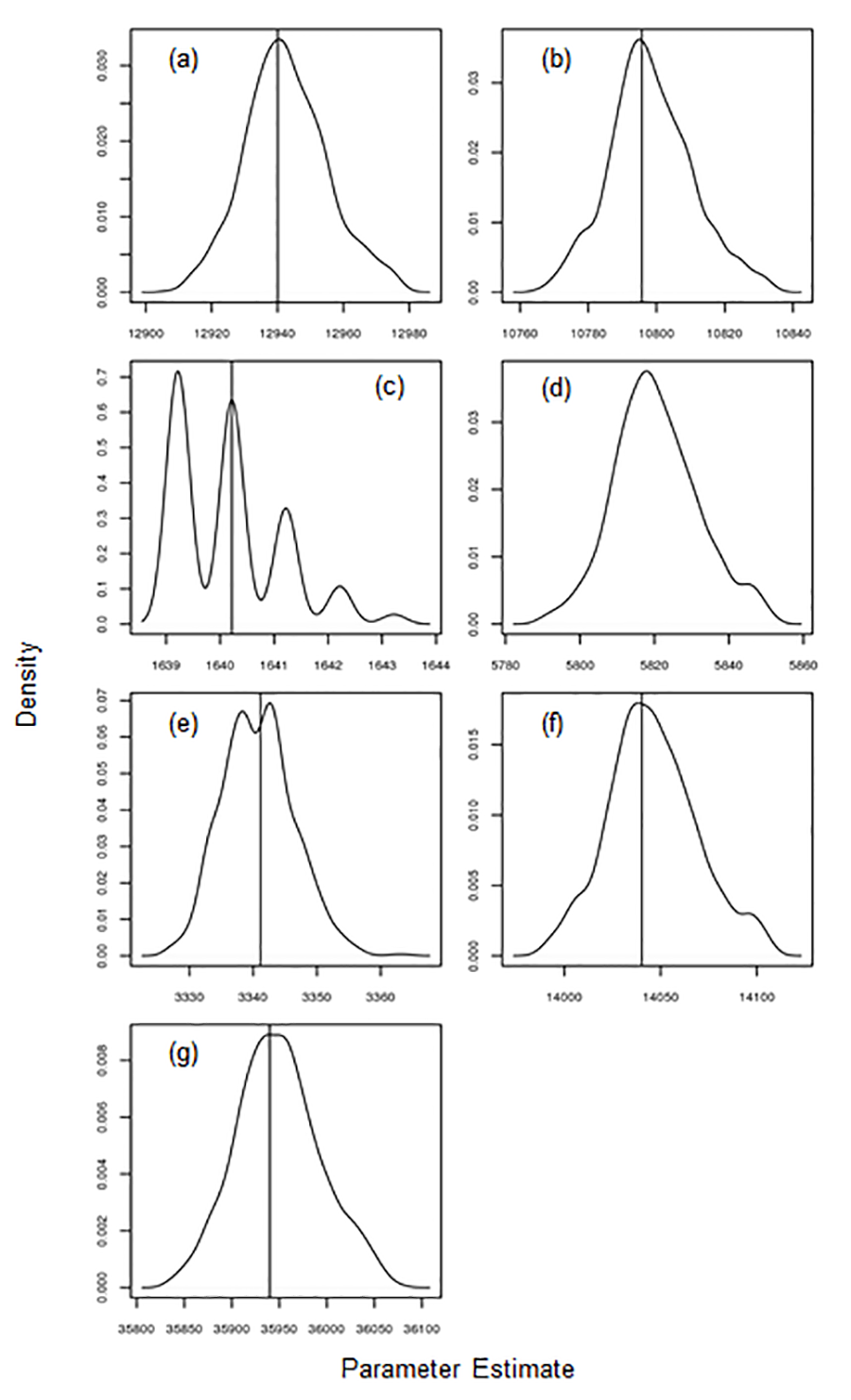

Supplement: S2 Fig — Each panel shows a histogram of the distribution of statistics for n = 1000 simulated models from the observed model. The vertical bar shows the observed statistic from the data. The best fit models have observed statistics near the center of the distribution of simulated statistics. Panels show the distributions for the terms (a) sum, (b) node match of sex, (c) node match of matriline, (d) node match of rank, (e) node factor of low rank, (f) node factor of medium rank, and (g) edge covariate of the log of the number of months of overlap. (TIF) [file pone.0215060.s002.tif]

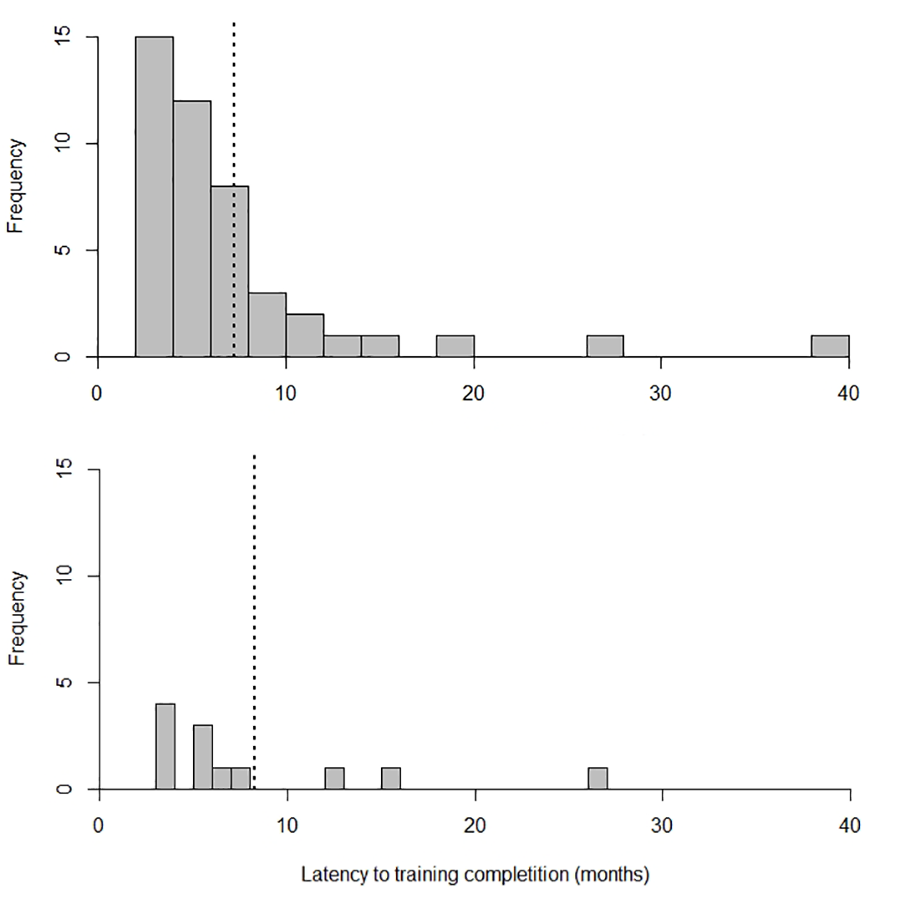

Supplement: S3 Fig — Females (top) and males (bottom). Dotted lines indicates mean latency. (TIF) [file pone.0215060.s003.tif]
