# Supplementary material for: Influences of demographic, seasonal, and social factors on automated touchscreen computer use by rhesus monkeys (Macaca mulatta) in a large naturalistic group
Source: PLoS One. 2019 Apr 24;14(4):e0215060. doi: 10.1371/journal.pone.0215060 (PMC6481812; doi:10.1371/journal.pone.0215060)
Supplement: S1 Table — All data present means ± standard deviations. (PDF) [file pone.0215060.s004.pdf]

**Task**	**Number of Monkeys Participating in the Task**	**Average Trial Latency (s)**	**Average Number of Trials Completed**	**Average Total Time Engaged in Task (s)**
Match to Sample	54	3.41 ± 0.16	10614.07 ± 2042.81	41418.9 ± 10248.79
Classification	35	3.03 ± 0.17	20861.69 ± 3161.60	52064.79 ± 7029.87
Perceptual Classification	13	1.71 ± 0.08	7196.69 ± 952.94	11937.93 ± 1473.93
Transitive Inference	14	1.29 ± 0.05	7205.71 ± 849.51	9252.99 ± 1133.59
Dominance Identification	10	5.79 ± 0.43	1550.40 ± 607.18	7502.72 ± 2686.61
Matriline Classification	13	7.57 ± 0.11	2030.31 ± 476.54	15503.63 ± 3688.54
