# Supplementary material for: Influences of demographic, seasonal, and social factors on automated touchscreen computer use by rhesus monkeys (Macaca mulatta) in a large naturalistic group
Source: PLoS One. 2019 Apr 24;14(4):e0215060. doi: 10.1371/journal.pone.0215060 (PMC6481812; doi:10.1371/journal.pone.0215060)
Supplement: S2 Table — (PDF) [file pone.0215060.s005.pdf]

Term	Estimate	Std. Error	Z Value	P value
Intercept	0.165	0.430	0.385	.700
Sex[1]	-1.425	0.645	-2.211	.027
Age at training	-0.020	0.006	-3.166	.002
Low ranking[2]	0.779	0.491	1.587	.113
Medium ranking[3]	1.581	0.504	3.138	.002
Sex*Age at training	0.003	0.024	0.107	.915

[1] 1: Male, 0: Female

[2] 1: Low-ranking, 0: Otherwise

[3] 1: Medium-ranking, 0: Otherwise
