# Supplementary material for: Influences of demographic, seasonal, and social factors on automated touchscreen computer use by rhesus monkeys (Macaca mulatta) in a large naturalistic group
Source: PLoS One. 2019 Apr 24;14(4):e0215060. doi: 10.1371/journal.pone.0215060 (PMC6481812; doi:10.1371/journal.pone.0215060)
Supplement: S3 Table — (PDF) [file pone.0215060.s006.pdf]

Term	Estimate	Std. Error	T Value	P value
Intercept	5.453	1.547	3.526	< .001
Sex[1]	3.390	1.537	2.206	.027
High ranking[2]	1.635	1.105	1.479	.139
Medium ranking[3]	1.933	0.927	2.086	.037
Age at training	0.030	0.020	1.522	.128
Age at testing	0.018	0.041	0.432	.666
Sex*Age at training	-0.028	0.042	-0.663	.507

[1] 1: Female, 0: Male

[2] 1: High-ranking, 0: Otherwise

[3] 1: Medium-ranking, 0: Otherwise

[4] Smoothed effect of month $F(6.442, 6.442) = 3.481$, $p < .001$

[5] Estimate of subject-specific random intercept variance: 7.910
