# Supplementary material for: Influences of demographic, seasonal, and social factors on automated touchscreen computer use by rhesus monkeys (Macaca mulatta) in a large naturalistic group
Source: PLoS One. 2019 Apr 24;14(4):e0215060. doi: 10.1371/journal.pone.0215060 (PMC6481812; doi:10.1371/journal.pone.0215060)
Supplement: S4 Table — (PDF) [file pone.0215060.s007.pdf]

Term	Estimate	Std. Error	T Value	P value
Intercept	9.564	1.501	6.374	< .001
Reproductive experience[1]	-1.438	0.415	-3.461	< .001
High ranking[2]	1.508	1.649	0.914	.361
Medium ranking[3]	2.599	1.352	1.922	.055
Age at training	0.042	0.026	1.604	.109
Age at testing	-0.013	0.014	-1.606	.109

[1] 1: Parous, 0: Nulliparous

[2] 1: High-ranking, 0: Otherwise

[3] 1: Medium-ranking, 0: Otherwise

[4] Smoothed effect of month $F(4.627, 9) = 3.192$, $p < .001$

[5] Estimate of subject-specific random intercept variance: 12.309
