# Supplementary material for: Influences of demographic, seasonal, and social factors on automated touchscreen computer use by rhesus monkeys (Macaca mulatta) in a large naturalistic group
Source: PLoS One. 2019 Apr 24;14(4):e0215060. doi: 10.1371/journal.pone.0215060 (PMC6481812; doi:10.1371/journal.pone.0215060)
Supplement: S5 Table — (PDF) [file pone.0215060.s008.pdf]

Term	Estimate	Std. Error	T Value	P value
Intercept	4.363	1.520	2.870	.004
High ranking[1]	-0.577	1.387	-0.416	.678
Medium ranking[2]	1.410	1.185	1.190	.234
Age at training	-0.218	0.064	-3.417	< .001
Age at testing	0.326	0.038	8.471	< .001

[1] 1: High-ranking, 0: Otherwise

[2] 1: Medium-ranking, 0: Otherwise

[3] Smoothed effect of month $F(1.77, 9) = 0.634$, $p = .028$

[4] Estimate of random subject-specific intercept variance: 5.164
