# Supplementary material for: Influences of demographic, seasonal, and social factors on automated touchscreen computer use by rhesus monkeys (Macaca mulatta) in a large naturalistic group
Source: PLoS One. 2019 Apr 24;14(4):e0215060. doi: 10.1371/journal.pone.0215060 (PMC6481812; doi:10.1371/journal.pone.0215060)
Supplement: S6 Table — (PDF) [file pone.0215060.s009.pdf]

Term	Estimate	Std. Error	T Value	P value
Intercept	7.122	0.619	11.506	< .001
Sex[1]	-0.923	0.870	-1.061	.289
Age at training	-0.016	0.015	-1.074	.283

[1] 1: Male, 0: Female

touchscreen[2] Smoothed effect of month for high ranking monkeys $F(4.621, 4.621) = 155.7$, $p < .001$

[3] Smoothed effect of month for medium ranking monkeys $F(4.775, 4.775) = 256.3$, $p < .001$

[4] Smoothed effect of month for low ranking monkeys $F(4.354, 4.354) = 115.2$, $p < .001$

[5] Estimate of random intercept variance: 6.113
