# Supplementary material for: Influences of demographic, seasonal, and social factors on automated touchscreen computer use by rhesus monkeys (Macaca mulatta) in a large naturalistic group
Source: PLoS One. 2019 Apr 24;14(4):e0215060. doi: 10.1371/journal.pone.0215060 (PMC6481812; doi:10.1371/journal.pone.0215060)
Supplement: S7 Table — (PDF) [file pone.0215060.s010.pdf]

Term	Estimate	Std. Error	P value
Sum	-5.571	0.700	< .001
Node match sex[1]	3.201	0.662	< .001
Node match matriline	-1.096	1.208	.364
Node match rank category	0.772	0.378	.042
Low ranking[1]	-0.923	0.410	.024
Medium ranking[2]	-0.670	0.376	.067
Edge covariate of log(overlap in group)	1.918	0.124	< .001

[1] 1: Female, 0: Male
[2] 1: Low-ranking, 0: Otherwise
[3] 1: Medium-ranking, 0: Otherwise
[4] Converged after 8 iterations
