# Supplementary material for: Influences of demographic, seasonal, and social factors on automated touchscreen computer use by rhesus monkeys (Macaca mulatta) in a large naturalistic group
Source: PLoS One. 2019 Apr 24;14(4):e0215060. doi: 10.1371/journal.pone.0215060 (PMC6481812; doi:10.1371/journal.pone.0215060)
Supplement: S1 File — (PDF) [file pone.0215060.s011.pdf]

S1 File. Social network description and diagnostics.

Model description

The valued exponential random graph model [58] included both endogenous (i.e. structural features of the network) and exogenous (i.e. related to properties of the nodes and edges) covariates. For endogenous covariates, we included the sum of the edge weights, which is analogous to an intercept in a generalized linear model. Exogenous covariates included a node factor of rank (which adds a term to the model for the total weight of the edges that each rank category contributes), a node match of rank (which counts the total weight of edges which are between like-ranked individuals), a node match of sex (which counts the total weight of edges between individuals of like-sex), a node match of matriline (which counts the total amount of weight between edges within the same matriline), and an edge covariate of the log of number of months (+.01 months) that animals spent in the group together (which controls for differential time that animals co-inhabited the group together). The model was seeded with a contrastive divergence (CD) procedure and estimated using a Monto Carle Maximum Likelihood Estimation (MCMLE). The MCMC interval and sample size were set at 1000 and the maximum number of iterations for MCMLE was set to 200.

Model evaluation

The convergence of the model was assessed using Geweke's diagnostic [74] on the MCMC chains, which suggested that all chains have converged (All p's > .3, where higher p values suggest convergent chains). Graphically, the run plots for the MCMC chains for each term showed random noise around a mean value, again suggesting convergent appropriately (S1 Fig).

This took 8 iterations of the MCMLE procedure. We also investigated the goodness of fit of the model by simulating networks (n = 1000) from the fit model. The statistics included in the model (sum, node match sex, node match rank, node match rank, node factor rank, and edge covariate of observation time) were calculated for each simulated network, and the distribution of simulated statistics was compared to the observed statistics. For most terms (S2 Fig), the network model accurately predicted the modelled statistics, making us confident that the model was appropriate for the data.

Reference

74. Geweke J. Evaluating the accuracy of sampling-based approaches to calculating posterior moments. In: Bernado JM, Berger JO, Dawid AP, Smith AFM, editors. Bayesian Statistics (Vol. 4). Oxford, UK: Clarendon Press. 1992.
